# The evolutionary significance of whole genome duplications in oil biosynthesis of oil crops

**DOI:** 10.1093/hr/uhae156

**Published:** 2024-06-07

**Authors:** Jingjing Wu, Linjing Zhang, Xiaohui Ma, Xinxing Fu, Fei Chen, Yuannian Jiao, Jianquan Liu, Shengdan Wu

**Affiliations:** State Key Laboratory of Herbage Improvement and Grassland Agro-ecosystems, College of Ecology, Lanzhou University, Lanzhou 730000, China; College of Life Sciences, Shanxi Normal University, Taiyuan 030031, China; State Key Laboratory of Herbage Improvement and Grassland Agro-ecosystems, College of Ecology, Lanzhou University, Lanzhou 730000, China; College of Life Sciences, Northwest Normal University, Lanzhou 730070, China; National Key Laboratory for Tropical Crop Breeding, Sanya Institute of Breeding and Multiplication, Hainan University, Sanya 572025, China; School of Tropical Agriculture and Forestry, Hainan University, Haikou 570228, China; State Key Laboratory of Plant Diversity and Specialty Crops, Institute of Botany, the Chinese Academy of Sciences, Beijing 100093, China; State Key Laboratory of Systematic and Evolutionary Botany, Institute of Botany, Chinese Academy of Sciences, Beijing 100093, China; University of Chinese Academy of Sciences, Beijing 100049, China; State Key Laboratory of Herbage Improvement and Grassland Agro-ecosystems, College of Ecology, Lanzhou University, Lanzhou 730000, China; State Key Laboratory of Herbage Improvement and Grassland Agro-ecosystems, College of Ecology, Lanzhou University, Lanzhou 730000, China

Dear Editor,

Humans cannot survive without plants because many plants have evolved beneficial traits that not only support their own survival and reproduction but also provide essential goods, including food, for humans. One such advantageous trait is the ability of certain plants, known as oil crops, to generate and accumulate a high content of oil, specifically triacylglycerol (TAG), in their seeds. For plants, storing a substantial amount of oil in seeds serves as an important energy source for seed germination and early seedling development. However, for humans, the oils extracted from these seeds have become an indispensable component of our daily diet and are also used as feedstocks in various industries. It is projected that there will be a rapid increase in global demand for vegetable oils, and the market for these oils is expected to reach approximately US$343.9 billion by 2028 (source: https://www.imarcgroup.com/vegetable-oil-processing-plant). This trend has incentivized significant efforts to discover more oil-yielding plants and improve the yield of crop oil.

Whole genome duplications (WGDs), also known as polyploidy, involve the doubling of an organism’s entire genome. These events have long been recognized as major drivers of genetic changes, potentially leading to significant evolutionary and ecological consequences [1]. For example, WGDs are believed to have played a crucial role in key evolutionary innovations [2], such as the emergence of flowers in angiosperms [3] and the development of glucosinolates in the cruciferous family [4]. However, the specific contribution of WGDs to oil biosynthesis in oil crops remains uncertain. In this study, we conducted a comprehensive analysis of this issue by assembling a high-quality genome of *Elaeagnus mollis* Diels (Elaeagnaceae), an oil crop, to investigate the origin of oil biosynthesis-related genes resulting from WGDs. Subsequently, we examined the overall impact of WGDs on genes associated with oil biosynthesis in other major oil crops.

**Table 1:** Statistics of genome assembly for *Elaeagnus mollis*

**Features**	**Statistics**
Length of genome assembly (Mb)	625.29
Contig N50 (Mb)	15.99
Longest Contig (Mb)	67.09
Scaffold N50 (Mb)	35.79
Longest Scaffold (Mb)	113.69
Anchored to chromosome (Mb)	600.51
Anchored ratio (%)	96.04
BUSCO score of assembly (%)	98.60

**Figure 1:**
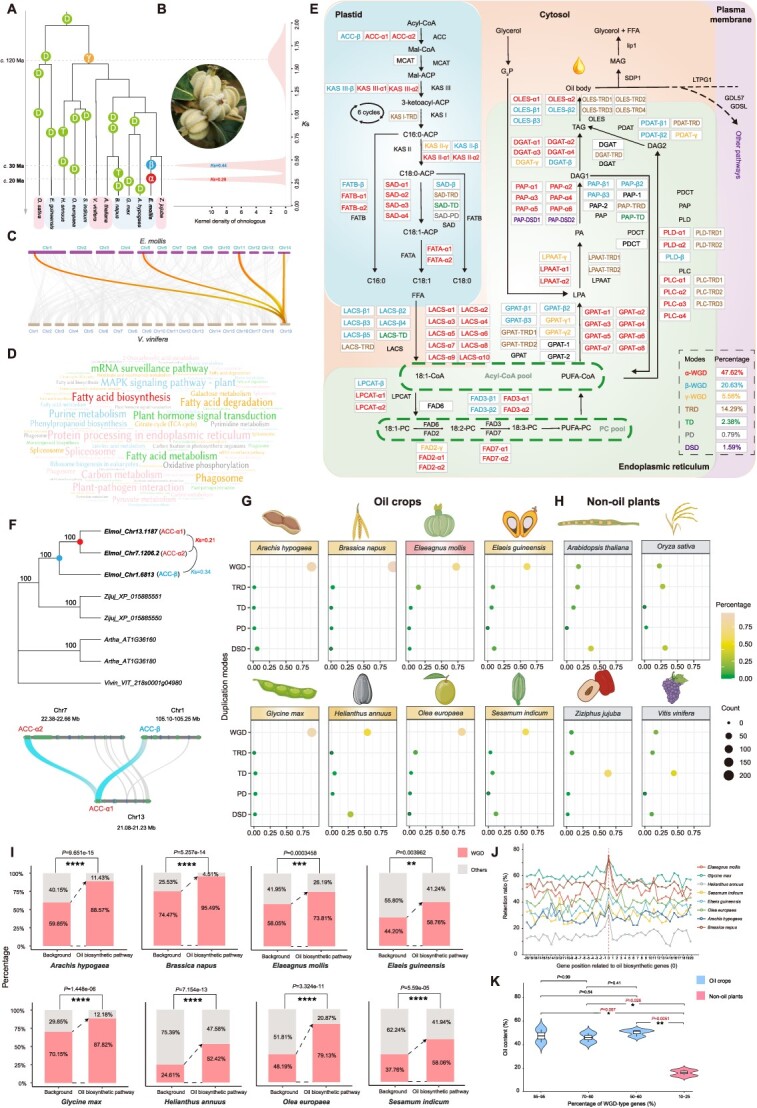
The evolutionary significance of WGDs in oil biosynthesis of oil crops. **A** Phylogenetic position of the WGDs in *Elaeagnus mollis* and other major oil crops. A photograph near the phylogeny showing the 8-ribbed fruit of *E. mollis* (Credit: Ze Wei, Institute of Botany, CAS). **B***K*s distributions of *E. mollis* paralogous. Three *K*s peaks corresponds to the α, β, and γ WGDs as shown in (**A**). **C** Synteny comparisons between the genomes of *E. mollis* and *Vitis vinifera*. One syntenic block in *V. vinifera* has four corresponding homologous regions in the *E. mollis* genome. **D** The wordcloud shows the enriched KEGG terms of the WGD-type genes in *E. mollis*. **E** Overview of the vegetable oil biosynthetic pathway [6, 7], along with the genes and their duplication modes in *E. mollis* involved in major reaction steps. Genes of different duplication modes are color-coded: red with -α tail label for genes derived from α WGD, blue with -β tail label for genes derived from β WGD, yellow with -γ tail label for genes derived from γ WGD, green with -TD tail label for genes derived from tandem duplications, brown with -TRD tail label for genes derived from transposed duplications, purple with -DSD tail label for genes derived from dispersed duplications, and gray with -PD tail label for genes derived from proximal duplications. Single-copy genes are represented in black font. The bottom right corner shows the proportion of genes of different duplication modes. Abbreviations: ACC, acetyl-CoA carboxylase; DGAT, diacylglycerol acyltransferase; FAD2 and FAD6, fatty acid Delta-12 desaturase; FAD3 and FAD7, fatty acid Delta-15 desaturase; FATA/B, acyl-ACP thioesterase A/B; GPAT, G3P acyltransferase; KAS I, ketoacyl-ACP synthase I; KAS II, ketoacyl-ACP synthase II; KAS III, ketoacyl-ACP synthase III; LACS, long-chain acyl-CoA synthetase; LPAAT, LPA acyltransferase; LPCAT, 1-acylglycerol-3-phosphocholine acyltransferase; MCAT, malonyl-CoA transacylases; PAP, phosphatidic acid phosphatase; PDAT, phospholipid-DAG acyltransferase; PDCT, phosphatidylcholine; PLC and PLD, phospholipase C and D; SAD, stearoyl-ACP desaturase. **F** Exemplar phylogeny of the ACC gene family shows the duplication events in *E. mollis*. Solid circles with red and blue colors indicate duplications derived from the corresponding α and β WGDs as in (**A**). Syntenic blocks with ACC genes were placed on the right of the phylogenetic tree. **G–H** The numbers and proportions of oil biosynthetic genes derived from different modes of duplication in eight oil crops and four non-oil plants. **I** Comparison of the percentage of WGD-derived duplicate genes in oil biosynthetic pathway with the proportion of duplicate genes generated by WGDs against all genes in the genome of each species. **J** The retention ratio of oil biosynthetic genes and their neighboring genes (the 20 genes to the left and the 20 genes to the right) after WGDs among eight oil crops. Different colored lines represent different species. The position of the red dashed line indicates the location of oil biosynthetic genes in different species. **K** The violin plots illustrate the correlation between the proportion of WGD-derived genes and the oil content in oil crops compared to non-oil plants. Student’s *t*-test was used for the significance test. The 85–95 group includes *Brassica napus* and *Arachis hypogaea*; the 70–80 group includes *Olea europaea* and *E. mollis*; the 50–60 group includes *Elaei guineensis*, *Sesamum indicum*, and *Helianthus annuus*; the 10–25 group includes *Oryza sativa* and *V. vinifera*.

The oil crop *E. mollis* exhibits a remarkable oil yield, with the seeds surpassing 50%, and the oil containing over 91% unsaturated fatty acids [5]. We successfully generated a high-quality genome for this diploid species (2n = 2x = 28). The assembled genome was 625 Mb in size, and we annotated a total of 33 446 putative protein-coding genes. The completeness of the genome assembly was assessed using BUSCO, yielding a completeness score of 98.60%, indicating high assembly quality (Table 1). Our analysis revealed two-consecutive ancient WGD events, named α and β, specific to this genome approximately 30 and 20 million years ago (Fig. 1A–C). We found that the duplicated genes from WGDs were over-represented in pathways related to fatty acid biosynthesis and metabolism (Fig. 1D). Oil biosynthesis involves two major processes: fatty acid biosynthesis in the plastids and TAG assembly in the endoplasmic reticulum [6, 7] (Fig. 1E). To identify the corresponding orthologs of oil biosynthesis genes in *E. mollis*, we used the well-characterized oil biosynthetic genes from the model plant Arabidopsis as a reference (ARALIP: http://aralip.plantbiology.msu.edu/). Surprisingly, we discovered that nearly 74% of the genes required for vegetable oil biosynthesis in *E. mollis* were WGD-derived duplicate genes (Fig. 1E). This percentage significantly exceeded the proportion of WGD-derived genes among all genes in the *E. mollis* genome (58%) (*P* = 0.0003458, Chi-square test). To further investigate the relative contribution of each WGD event to oil biosynthesis, we classified the corresponding WGD-derived gene duplicates in *E. mollis* based on a combination of phylogenetic, synteny, and *K*s analyses [8]. Both phylogenetic analyses and the *K*s values enabled us to distinguish between paralogous genes from the α and β WGD (Fig. 1E–F) that are involved in vegetable oil biosynthetic pathways. For example, among the three members of the ACC family, which initiates fatty acid biosynthesis by converting acetylCoA and bicarbonate into malonyl-CoA, two (ACC-α1 and ACC-α2) are retained from the α WGD, and the other (ACC-β) is derived from the β WGD (Fig. 1F). Overall, our findings demonstrate that two specific WGDs have played a significant role in expanding the gene repertoire, accounting for 60 and 26 out of the total 126 genes involved in vegetable oil biosynthesis in *E. mollis* (Fig. 1E).

We proceeded to investigate whether the significant contribution of WGDs to oil biosynthesis, as observed in *E. mollis*, was also prevalent in other oil crops using similar approaches. The DupGene_finder pipeline (https://github.com/qiao-xin/DupGen_finder) was used to classify the different modes of gene duplications. We examined the gene duplication patterns of oil biosynthetic genes in seven well-known oil crops with published genomes, namely peanut (*Arachis hypogaea*), rapeseed (*Brassica napus*), oil palm (*Elaeis guineensis*), soybean (*Glycine max*), sunflower (*Helianthus annuus*), olive (*Olea europaea*), and sesame (*Sesamum indicum*) (Fig. 1G). Consistently, the results obtained from various oil crops revealed a clear trend where duplicate genes derived from WGDs constituted the majority of members in the oil biosynthetic pathway. The proportion of WGD-derived genes varied, ranging from 52% in sunflower to approximately 95% in rapeseed (Fig. 1G). In comparison, we also examined four non-oil plants and observed distinct patterns compared to oil crops (Fig. 2H). For instance, dispersed duplications (DSD) accounted for the largest proportion, approximately 38% and 28%, of the vegetable oil biosynthetic genes in *Arabidopsis thaliana* and *Oryza sativa*, respectively. In *Vitis vinifera* and *Ziziphus jujube*, duplicates mediated by tandem duplications (TD) contributed the most, at 45% and 63%, respectively, to the oil biosynthetic genes (Fig. 2H). Notably, the contribution from WGDs in these non-oil plants was limited, ranging from 7% in *Z. jujube* to 17% in *A. thaliana*. The contrasting patterns observed between oil crops and non-oil plants provide compelling evidence that WGDs, as the primary source of oil biosynthetic genes in oil crops, have played a recurring role in driving oil biosynthesis in diverse plants that were subsequently domesticated by humans.

The substantial contribution of WGDs to oil biosynthesis in oil crops may be explained by strong selection for oil-related traits following WGD events. These crops have also experienced paleopolyploidy throughout their evolutionary history (Fig. 1A) [9]. While WGD-derived duplicate genes are not specific to the oil biosynthesis pathway, they have undergone preferential retention in this pathway. For example, in *A. hypogaea* and *B. napus* genomes, WGD-derived duplicate genes occupy 59.85% and 74.47%, respectively, of the total genes, but their proportion significantly increases to 88.57% and 95.49% in the oil biosynthesis pathway (Fig. 1I). Similar patterns are observed in the other six main oil crops, where WGD-derived duplicate genes are significantly and preferentially retained in the oil biosynthetic pathway compared to the backgrounds. (Fig. 1I). Furthermore, we analysed the retention of oil biosynthetic genes and their neighboring genes in eight oil crops. The results revealed that, in comparison to neighboring genes, the retention ratio of most oil biosynthetic genes was substantially higher (Fig. 1J). The retention of duplicated genes from WGDs can lead to the development of novel regulatory relationships through processes like neo-functionalization and sub-functionalization, resulting in evolutionary innovations [1–4, 8, 10]. Therefore, within this evolutionary framework, the preferentially retained duplicated genes in oil biosynthetic pathways following WGDs likely contribute to genetic innovations underlying oil biosynthesis across diverse lineages. As expected, we observed that oil crops with a high percentage of WGD-derived genes in their oil biosynthesis pathway generally have significantly higher oil content compared to non-oil plants (Fig. 1K). Therefore, these investigated oilseed crops from different lineages seem to commonly take advantage of WGDs for generating their oil biosynthesis pathways. Overall, our research underscores the importance of WGDs in generating crucial agronomic traits that have shaped human diet and civilization.

## Author contributions

S.W., J.L., Y.J., and F.C. designed the research; J.W., L.Z., and S.W. performed the research; J.W. X.F., X.M., and S.W. analysed the data; and S.W., J.L., Y.J., and F.C. wrote the paper. All authors read and approved its content.

## Data availability

The genome assembly and annotation of *E. mollis*, as well as the relevant supporting information, are available on the Figshare website (https://figshare.com/s/49297f3d432c0f179ef7). Additional data necessary to reproduce the analyses presented in this study are available from the corresponding authors upon reasonable request.

## Conflict of interest statement

The authors declare no conflict of interest.
